# Risk of incident atrial fibrillation in patients presenting with retinal artery or vein occlusion: a nationwide cohort study

**DOI:** 10.1186/s12872-018-0825-1

**Published:** 2018-05-10

**Authors:** Christine Benn Christiansen, Christian Torp-Pedersen, Jonas Bjerring Olesen, Gunnar Gislason, Morten Lamberts, Nicholas Carlson, Mathias Buron, Nikolai Juul, Gregory Y. H. Lip

**Affiliations:** 1 Aalborg University Hospital, Denmark, Søndre Skovvej 15, 9000 Aalborg, Denmark; 2 Department of Health Science and Technology, Aalborg University, Fredrik Bajers Vej 7D, 9220 Aalborg, Denmark; 3 Department of Cardiology, Gentofte Hospital, University of Copenhagen, Kildegårdsvej 28, 2900 Hellerup, Denmark; 4 National Institute of Public Health, University of Southern Denmark, Øster Farimagsgade 5A, Copenhagen, Denmark; 5 Faculty of Health and Medical Sciences, University of Copenhagen, Copenhagen, Denmark; 6 University of Birmingham Centre for Cardiovascular Sciences, City Hospital, Edgbaston, Birmingham, Birmingham, B15 2TT UK; 7 The Danish Heart Foundation, Vognmagergade 7, 3. sal, 1120 Copenhagen, Denmark

**Keywords:** Epidemiology, Retinal artery occlusion, Retinal vein occlusion,, Atrial fibrillation

## Abstract

**Background:**

The inter-relationships of atrial fibrillation (AF) to retinal vascular occlusions (whether retinal artery occlusion (RAO) or retinal venous occlusion (RVO)) remain unclear. It is unknown if a presentation of retinal artery or venous occlusions may indicate a new onset cardiac arrhythmia. To shed light on this association, we investigated the risk of new onset AF in patients with known RAO and RVO.

**Methods:**

Patients with retinal occlusions from 1997 to 2011 were identified through Danish nationwide registries and matched 1:5 according to sex and age. Cumulative incidence and unadjusted rates of AF according to retinal vascular occlusions (i.e. RAO or RVO) were determined. Hazard ratios (HR) of AF according to retinal vascular occlusion were adjusted for hypertension, diabetes, vascular disease and prior stroke/systemic thromboembolism/transient ischemic attack.

**Results:**

One thousand three hundred sixty-eight cases with retinal vascular occlusions were identified (median age 71.4 (inter quartile range (IQR); 61.2–79.8), 47.3% male). RAO constituted 706 cases (51.6%) and RVO 529 (38.7%). The rate of incident AF amongst all cases with retinal vascular occlusion was 1.74 per 100 person-years (95% confidence interval (CI), 1.47–2.06) compared to 1.22 (95% CI, 1.12–1.33) in the matched control group. The rate of AF in RAO was 2.01 (95% CI, 1.6–2.52) and 1.52 (1.15–2.01) in RVO. HRs of incident AF adjusted for cardiovascular comorbidities were 1.26 (95% CI; 1.04–1.53, *p* = 0.019) for any retinal vascular occlusion, 1.45 (95% CI; 1.10–1.89, *p* = 0.015) for RAO, and 1.02 (95% CI; 0.74–1.39, *p* = 0.920) for RVO.

**Conclusions:**

A new diagnosis of retinal vascular occlusion in patients without prior AF was associated with increased risk of incident AF, particularly amongst patients with RAO. Awareness of AF in patients with retinal vascular occlusions is advised.

## Background

Retinal artery and vein occlusions (RAO and RVO) are common causes of retinal vascular disease. The pathogenesis of RVO is mainly considered to be thrombus formation, whereas embolisms result in RAO. [1] Atrial fibrillation (AF) is not included amongst the known risk factors for RVO. [2] The embolic source in patients with RAO is thought to be the carotid artery or valvular disease, but patients with AF are at increased risk of developing RAO. [1, 3] Both conditions are associated with cardiovascular risk factors, such as hypertension and diabetes mellitus. [4–9] Similarly, patients with RAO and RVO are at increased risk of thrombosis-related complications such as myocardial infarction and stroke as well as silent brain infarction. [2, 10–16]

Ischemic stroke is an episode of neurological dysfunction caused by focal cerebral, spinal, or retinal infarction. [17] This definition of ischemic stroke applies to current guidelines, and implies that the work-up of patients with retinal vascular occlusions (i.e. RAO or RVO) should include a careful evaluation of cardiovascular risk factors. [6, 18, 19] While it has been argued that embolisms arising in the atria are too large to cause RAO, [20] valvular heart disease, as well as AF, may be risk factors for RAO. [11, 21–23] As AF is often asymptomatic, subclinical (silent) AF could represent the underlying cause in RAO patients without known AF,

Guidelines recommend treatment with anticoagulation to decrease the risk of ischemic stroke in patients with AF according to the CHA_2_DS_2_-VASc (congestive heart failure, hypertension, age > 75 years, diabetes, stroke, vascular disease, age 65–75 and female sex score). [24–26]^,^. We have previously shown that retinal vascular occlusion independently increases the risk of ischemic stroke and thromboembolism in patients with AF. [27] Accordingly, retinal vascular occlusion in patients with AF may merit treatment with anticoagulation.

We hypothesized that in patients without previously known AF, retinal vascular occlusions (i.e. RAO or RVO) overall would be associated with increased risk of incident AF. To explore these associations, we identified all new cases of retinal vascular occlusion in patients without prior AF in Denmark between 1997 and 2011 in a nationwide cohort study. Furthermore, independent subgroup analyses were performed for RAO and RVO.

## Methods

### Registers

Data was collected from the following Danish nationwide registers: The Danish National Patient Register contains information regarding all hospital admissions since 1974 classified by diagnoses according to International Classification of Diseases (ICD). ICD-8 codes were used from 1974 to 1995, and ICD-10 codes from 1996 onwards. [28] In the Register of Medicinal Product Statistics, all data on dispensed prescription drugs have been collected since 1994 coded according to The Anatomical Therapeutic Chemical (ATC) Classification System. [29, 30] Information regarding death and cause of death was accessed via the Danish Cause of Death Register. [31] Finally, linkage of data between registers is possible due to the unique identification number afforded all Danish residents.

### Definition of retinal vascular occlusion, comorbidities and outcome

RAO was identified according to ICD-8 code 37702 and ICD-10 code H340, H341 and H342. RVO was identified according to ICD-8 37703 and ICD-10 H348. Unspecified retinal vascular occlusion was defined by ICD-10 code H34 without specification of RAO or RVO. AF was identified by ICD-8 code 42793,42794 and ICD-10 code I48.

Other comorbidities established from discharge diagnosis were heart failure (ICD-8: 425, 4270, 4271, ICD-10:I110,J819, I42 and I50). Prior stroke, transitory ischemic attack or systemic thromboembolism (ICD-8: 433,434,435,436,444, ICD-10: I63, I64DG458, G459 and I74) and vascular disease (myocardial infarction and peripheral vascular disease (ICD-8: 410, 440, ICD-10: I21, I22, I700, I702, I703, I704, I705, I706, I707, I708 and I709).

Preceding use of anticoagulation was identified as redeemed prescriptions of warfarin (ATC: B01AA) prior to index date i.e. the date of the qualifying diagnosis of retinal vascular occlusions for cases. Index date for controls were the same date as for the corresponding cases. Medication at baseline was defined as redeemed prescriptions 180 days prior to admission. Prescriptions of any glucose-lowering drug (ATC: A10) defined diabetes mellitus. Specific drugs identified included; clopidogrel (ATC: B01AC04), dipyridamole (ATC: B01AC07), non-steroidal anti-inflammatory drugs (ATC: M01A except M01AX05), loop diuretics (ATC: C03C) and acetylsalicylic acid (ATC: B01AC06 and N02BA01). Notably, although acetylsalicylic acid can be sold over-the-counter, most use continues to be registered due to reimbursement policies.

Hypertension was defined as concurrent treatment with two antihypertensive drugs of different classes: antiadrenergic (ATC: C02A, C02B, C02C), non-loop diuretics (ATC: C02L, C03A, C03B, C03D, C03E, C03X, C07B, C07C, C07D, C08G, C02DA, C09BA, C09DA and C09XA52), vasodilators (C02DB, C02DD and C02DG), beta blockers (C07A, C07B, C07C, C07D and C07F), calcium channel blockers (C08, C07F, C09BB and C09DB), and renin angiotensin system inhibitors (ATC: C09AA, C09BA, C09BB, C09CA, C09DA, C09DB, C09XA02 and C09XA52). This method has previously been validated. [26]

### Study population and outcomes

All patients with discharge diagnosis of retinal vascular occlusions (i.e. RAO and RVO) from January 1st 1997 to December 31st 2011 were identified. Patients with previous AF or previous use of warfarin were excluded. Inclusion in the study happened on the day of discharge of retinal vascular occlusion. Each patient with retinal vascular occlusion was matched according to sex and age with five controls from the Danish National Patient Register using the Greedy Matching Macro (http://www.mayo.edu/research/documents/gmatchsas/doc-10027248). Controls were free from previous AF or prior use of warfarin. Patients were followed until incident of AF, death, or end of study period.

### Statistical analysis

Rates of AF were calculated as new cases of AF per 100 person-years. Cumulative incidence was calculated taking into account the competing risk of death. Risk of AF according to retinal vascular occlusion was determined by Cox regression analysis and presented unadjusted and adjusted for hypertension, diabetes, vascular disease, and prior stroke/systemic thromboembolism/transitory ischemic attack. Independent subgroup analyses were performed for RAO, RVO, and unspecified occlusions, using the corresponding controls as reference. Fulfillment of all model assumptions including proportional hazards, goodness of fit chi-square test, and significance of interactions between all covariates adjusted for in the model were tested and found valid.. Data management and statistical analyses were performed using SAS 9.2 (SAS Institute Inc., Gary, NC, USA) and R 3.0.2. (R Core Team (2013). R: A language and environment for statistical computing. R Foundation for Statistical Computing, Vienna, Austria. URL https://www.R-project.org).

### Ethics

Retrospective register based studies do not require ethical approval in Denmark and the study was approved by the Danish Data Protection Agency (Ref. no. 2007–58-0015 /Internal ref. no. GEH-2014-013, I-Suite number: 02731).

## Results

**Fig. 1:**
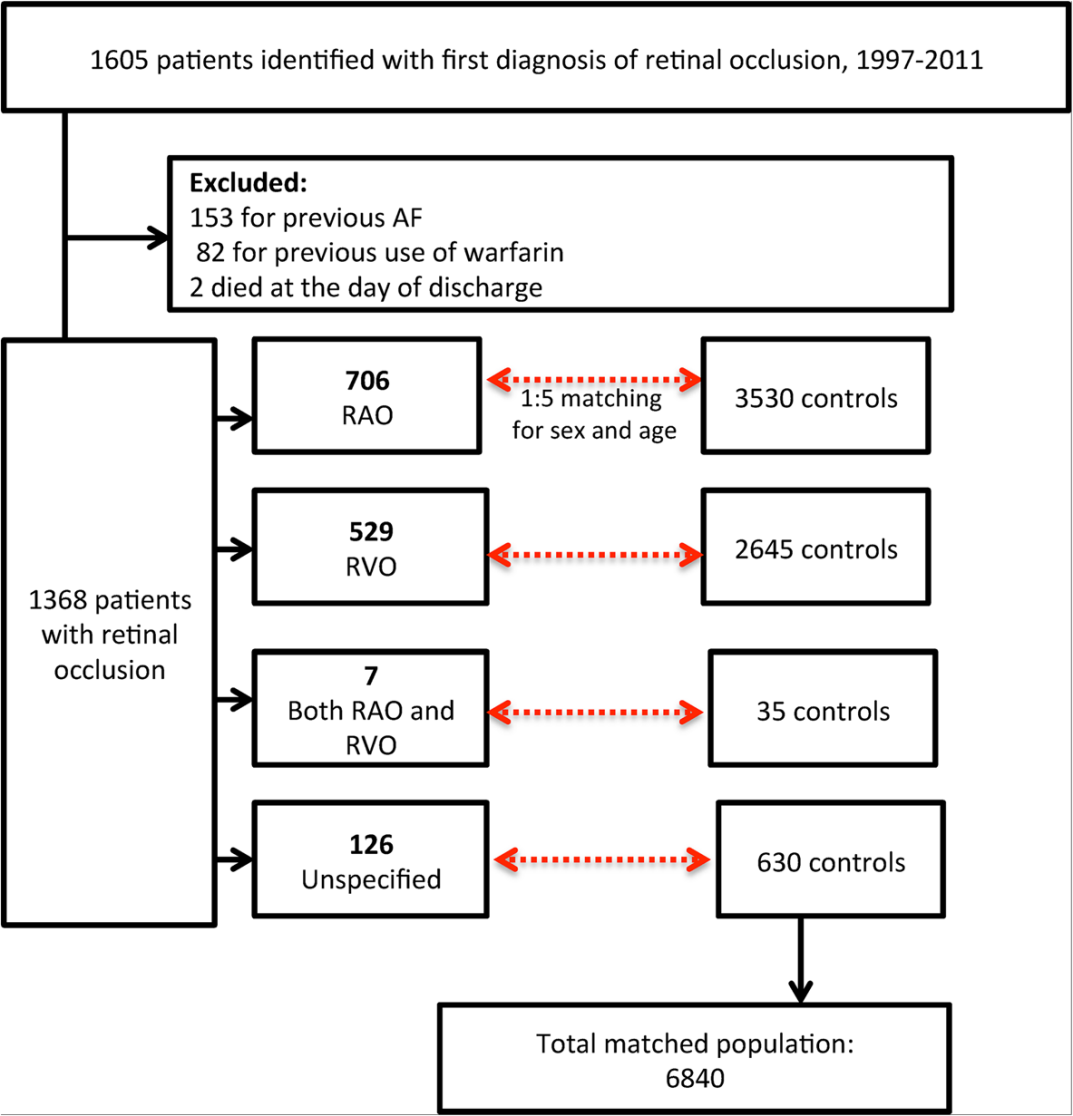
Flowchart of study population

Selection of the study population is depicted in Fig. 1. After exclusion of patients with prior diagnosis of AF and patients with preceding use of warfarin 1368 patients with retinal vascular occlusions remained; of these 706 (51.6%) had RAO and 529 (38.7%) had RVO. The number of patients with an unspecified diagnosis of retinal vascular occlusion (including those with both diagnosis of RAO and RVO on the day of inclusion) was 133 (9.7%).

The number of controls matched for sex and age was 6840. Baseline characteristics according to presence and type of retinal vascular occlusion are summarized in Table 1. The median age and gender distribution amongst cases and controls was 71.4 (interquartile range (IQR) 61.2–79.8) years and 47.3% were males. Comparably, patients with retinal vascular occlusion had more comorbidities and greater burden of cardiovascular medication compared to controls. Patients with RAO were slightly younger (median age 70.7 years (IQR 61.2–78.7)) and proportionately more male (49.7%) compared to patients with RVO (median age 73.4 years (IQR 62.3–81.3), 45.5% males). Furthermore, RAO patients more frequent had a history of stroke/transitory ischemic attack/systemic thromboembolism, as reflected in the use of clopidogrel and dipyridamole.

**Table 1:** Baseline characteristics of matched population

	Overall			Retinal arterial and vein occlusion		
	With retinal vascular occlusion	Controls	*p*-value	Arterial	Vein	*p*-value for difference between retinal artery and vein occlusion
n	1368	6840		706	529	
Median age, years (IQR)	71.4 (61.2–79.8)	71.4 (61.2–79.8)	*	70.7 (61.2–78.7)	73.4 (62.3–81.3)	< 0.01
Male	648 (47.3%)	3240 (47.3%)	*	351 (49.7%)	241 (45.6%)	0.150
Diabetes	115 (8.4%)	368 (5.4%)	< 0.01	59 (8.4%)	50 (9.5%)	0.502
Heart failure	67 (4.9%)	168 (2.5%)	< 0.01	34 (4.8%)	29 (5.5%)	0.599
Hypertension	391 (28.6%)	1188 (17.4%)	< 0.01	183 (25.9%)	160 (30.2%)	0.093
Stroke/TE/TIA	257 (18.8%)	395 (5.8%)	< 0.01	161 (22.8%)	62 (11.7%)	< 0.01
Vascular disease	148 (10.8%)	416 (6.1%)	< 0.01	92 (13%)	45 (8.5%)	0.012
Acetylsalicylic acid	514 (37.6%)	1236 (18.1%)	< 0.01	277 (39.2%)	190 (35.9%)	0.234
Antiadrenergic	24 (1.8%)	61 (0.9%)	< 0.01	10 (1.4%)	13 (2.5%)	0.181
Beta blockers	234 (17.1%)	753 (11%)	< 0.01	113 (16%)	94 (17.8%)	0.412
Calcium channel blockers	279 (20.4%)	831 (12.1%)	< 0.01	138 (19.5%)	107 (20.2%)	0.767
Clopidogrel	22 (1.6%)	55 (0.8%)	< 0.01	18 (2.5%)	3 (0.6%)	< 0.01
Loop diuretics	185 (13.5%)	590 (8.6%)	< 0.01	97 (13.7%)	77 (14.6%)	0.683
NSAID	235 (17.2%)	915 (13.4%)	< 0.01	125 (17.7%)	84 (15.9%)	0.397
Non-loop diuretics	405 (29.6%)	1347 (19.7%)	< 0.01	182 (25.8%)	170 (32.1%)	0.014
Dipyridamole	79 (5.8%)	108 (1.6%)	< 0.01	54 (7.6%)	16 (3%)	< 0.01

*=not calculated; age and sex were parameters used for matching, hence the distribution between was similar. *IQR* Interquartile range

The rates of incident AF are shown in Table 2. The rate of incident AF was higher in patients with any retinal vascular occlusion compared with patients without retinal vascular occlusion (1.74 incidents cases of AF per 100 person-years (95% confidence interval (CI); 1.47–2.06) versus 1.22 (95% CI; 1.12–1.33)). Incident AF rate according to type of occlusion was greater in the group with RAO (2.01 (95% CI; 1.6–2.52)) compared to patients with RVO (1.52 (95% CI; 1.15–2.01)).

**Table 2:** Crude rates of AF per 100 person-years according to retinal vascular occlusion

	With retinal vascular occlusion		
	No of patients	No of events	Rate of AF (95% confidence interval)
Any retinal vascular occlusion	1368	135	1.74 (1.47–2.06)
No retinal vascular occlusion (total matched control population)	6840	537	1.22 (1.12–1.33)
RAO	706	74	2.01 (1.6–2.52)
RVO	529	49	1.52 (1.15–2.01)
Unspecified retinal vascular occlusion	133	12	1.45 (0.82–2.55)

**Fig. 2:**
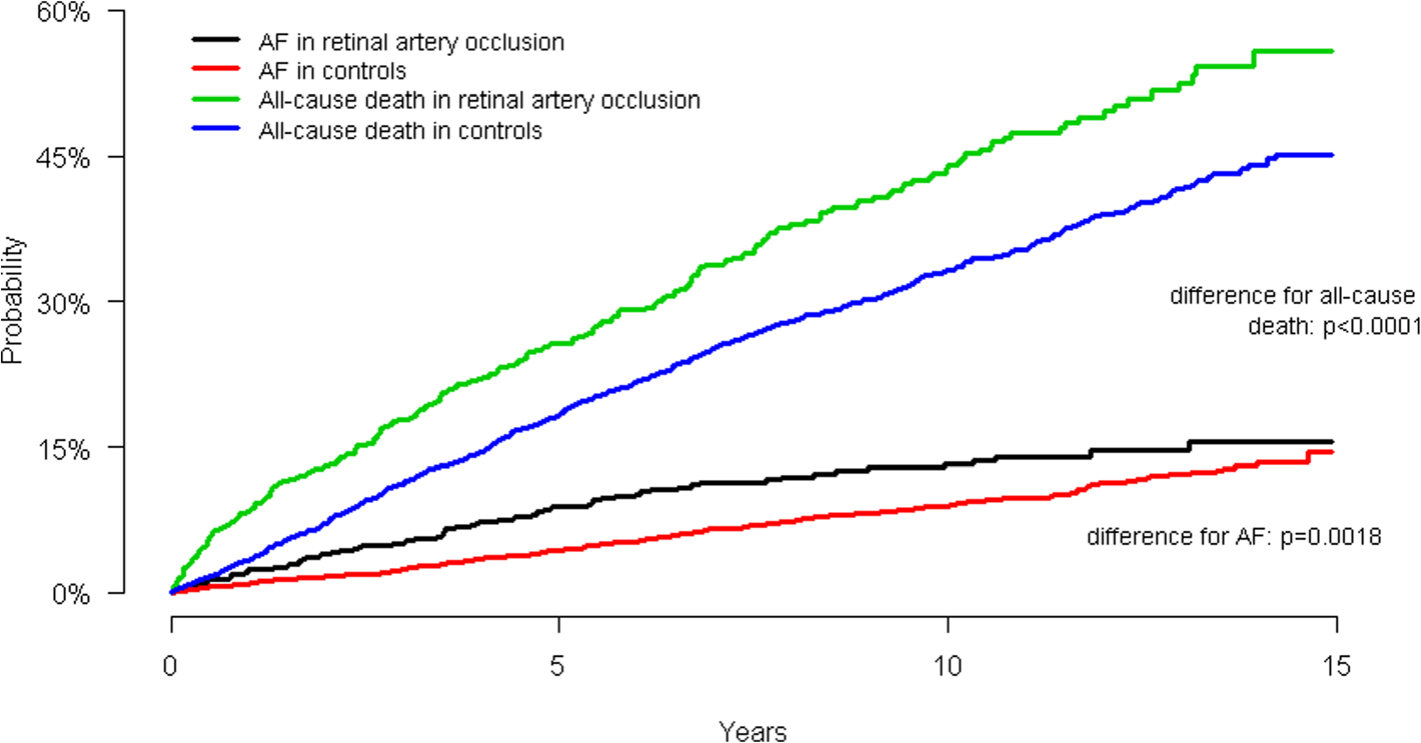
Cumulative incidence of atrial fibrillation and all-cause death according to retinal artery occlusion

**Fig. 3:**
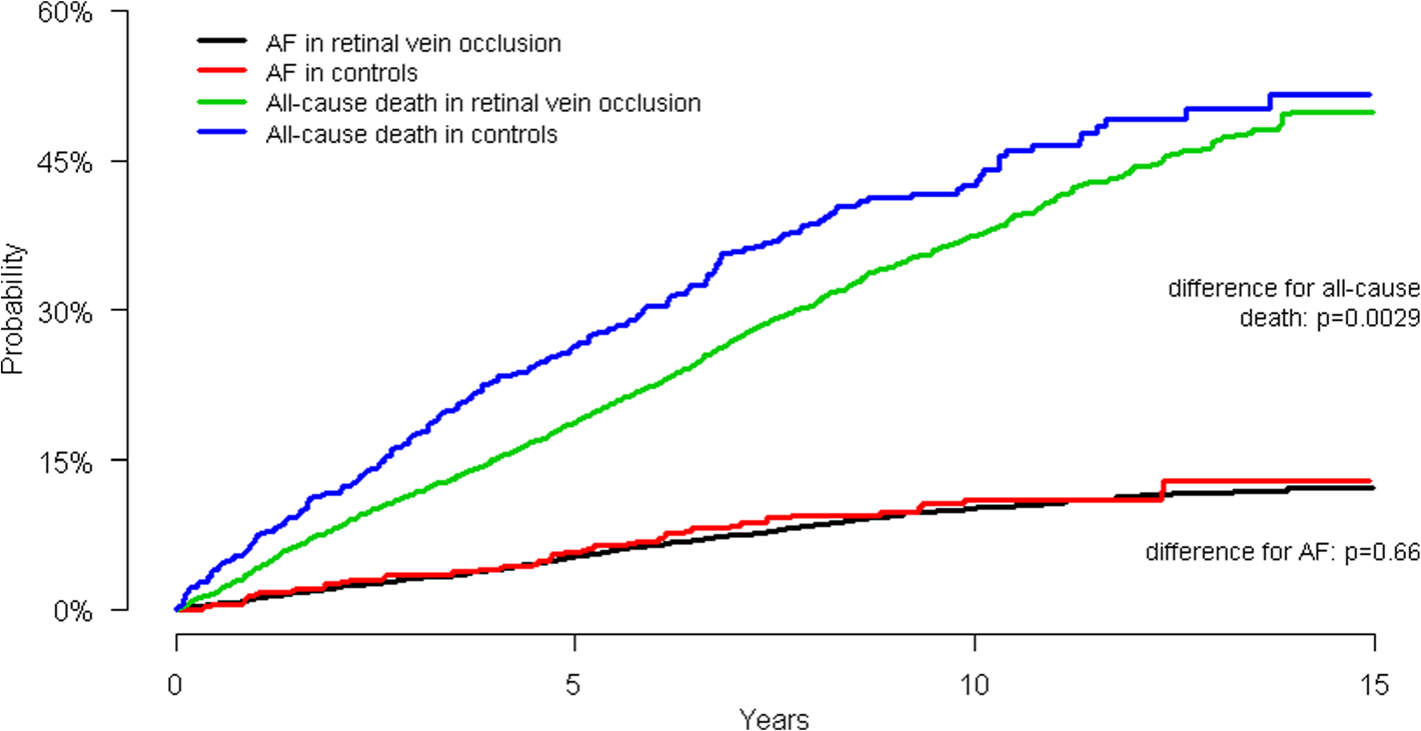
Cumulative incidence of atrial fibrillation and all-cause death according to retinal vein occlusion

Figures 2 and 3 show the cumulative incidence of AF and all-cause death in patients with RAO and RVO, respectively. The cumulative incidence of AF was significantly higher amongst patients with RAO compared with controls (*p* = 0.029), while no significant difference was observed between patients with RVO and the control group (*p* = 0.66). Cumulative incidence of all-cause death was also significantly different between RAO and matched controls (*p* <  0.001) and RVO and matched controls (*p* = 0.018).

**Fig. 4:**
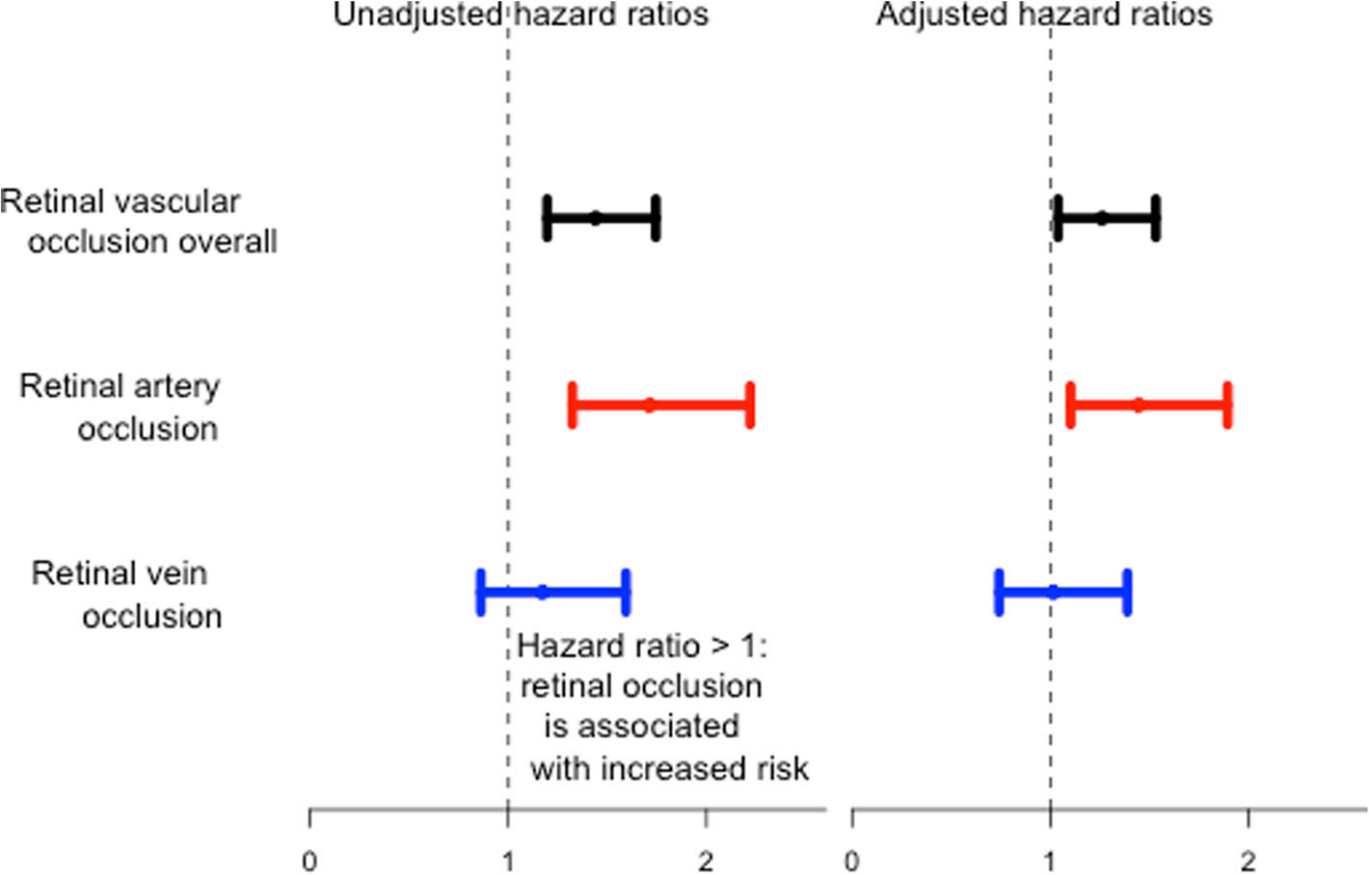
Hazard ratio of AF according to retinal vascular occlusion shown as unadjusted as well as adjusted for hypertension, diabetes, vascular disease and prior stroke/systemic thromboembolism/transitory ischemic attack. Reference is the corresponding controls without retinal vascular occlusion

Results from Cox regression analysis are shown in Fig. 4. The unadjusted hazard ratio (HR) of AF according to any retinal vascular occlusion was 1.44 (95% CI; 1.20–1.74, < 0.001), and the HR adjusted for heart failure, hypertension, diabetes, ischemic stroke/systemic thromboembolism/transitory ischemic attack and vascular disease the HR was 1.26 (95% CI; 1.04–1.53, *p* = 0.019).

In the subgroup analyses we calculated an unadjusted HR of AF amongst patients with RAO of 1.71 (95% CI; 1.32–2.22, p <  0.001) and adjusted HR of 1.45 (95% CI; 1.10–1.89, *p* = 0.015). For RVO the HRs of AF were non-significant (1.17 (95% CI; 0.86–1.59, *p* = 0.315) and (1.02 (95% CI; 0.74–1.39, *p* = 0.923) for unadjusted and adjusted analysis, respectively).

Patients with unspecified retinal vascular occlusion (including those diagnosed with coinciding artery and vein occlusions) had a rate of incident AF of 1.45 (95% CI; 0.82–2.55), unadjusted HR of 1.40 (95% CI; 0.74–2.65, *p* = 0.296) and an adjusted HR of 1.32 (0.68–2.59, *p* = 0.412).

## Discussion

In this register-based nationwide study of 1368 patients with a first-time diagnosis of retinal vascular occlusion, we found an increased risk of incident AF among patients with RAO, but not in patients with RVO. Given that AF may have been undiagnosed at the time of RAO, this strengthens the hypothesis that AF may in some cases be the underlying cause of RAO.

### Detection of AF

Etiologies for ischemic strokes can be classified as large-artery atherosclerosis, small-vessel occlusion, other determined etiology, undetermined etiology or cardioembolism according to the TOAST (Trial of Org 10,172 in Acute Stroke Treatment) criteria. [32] Cardio-embolism is estimated to be the source of stroke in 16–30% of all ischemic strokes, and 61% in patients with AF.

In stroke patients, guidelines recommend timely diagnosis of AF is prioritized to ensure prescription of oral anticoagulation to prevent future ischemic strokes. As AF in many cases is paroxysmal, detection rate of AF after ischemic stroke depends on the applied diagnostic method. Using a 7-day event-recorder, AF can be detected in 12.5–15% of stroke patients free of previous – known – AF. [33, 34] The detection rate increases further with continuous implantable and external cardiac monitoring. [35, 36]

The association of AF and RAO is not investigated to the same extent as the association of AF and ischemic stroke. Whereas one study found a similar detection rate of AF in RAO patients AF (12.5% of patients using 24-h ECG (at least)), other studies indicate that AF is more common in patients with ischemic stroke and find a higher number needed to screen in patients with RAO than in patients with ischemic stroke [14, 37, 38]. Patients with AF have a higher ratio of hemispheric to retinal occlusions compared to patients with AF, which may indicate that other embolic sources than the atria such as a carotid stenosis play a larger role in RAO patients. [39]

No studies have however investigated the proportion of cardio-embolic strokes in RAO. [40–42] In our population, less than 10% of the RAO patients were diagnosed with AF during follow-up. Several studies suggest assessment of cardiovascular risk in RAO could reasonably comprise Holter monitoring and trans-esophageal echocardiography. [21, 23, 33, 43] A clinical trial of patients with implanted devices capable of detecting silent episodes of AF would contribute to furthering knowledge on the association of AF with subsequent risk of retinal vascular occlusion.

We have previously shown how retinal vascular occlusion (either RAO or RVO) is an independent and substantial risk factor for stroke and thromboembolism in patients with known AF. In this study of 87,000 patients with AF, HR of RAO and RVO was 1.39 (1.08–1.79) and 1.26 (1.02–1.54), respectively, after adjustment for cardiovascular comorbidities and medication. [27]

In contrast to results from the present study, findings from another study found an increased risk of AF in patients with RVO, which may be explained by difference between the study designs or ethnicity of the populations. [44] Because RVO share risk factors with ischemic stroke, RVO may be interpreted as a stroke surrogate marker. [16] As such, AF detection in RVO may still be relevant especially since AF commonly develops asymptomatically, and the silent development of incident AF in patients with prior retinal vascular occlusions would infer greater risk of stroke in the absence of anticoagulation.

Since this is a registry-based cohort study, the results represent an association between the two conditions rather than a causal relation. The hypothesis that subclinical AF may be an underlying cause of RAO is supported by the significant increase in cumulative incidence of AF amongst RAO patients compared to controls. Comparably, no significant difference was found between RVO and controls. This observed difference is also relevant when addressing the issue of detection bias: Because this is an observational study, patients with retinal vascular occlusion conceivably receive greater medical attention after study inclusion compared with controls (i.e. surveillance bias), but the cumulative incidence of AF did not differ between RVO patients and controls, thereby countering the premise of surveillance bias. Thus, the observed results support a hypothesis of RAO, and not RVO, being caused by – undiagnosed – silent AF.

As we did not have access to information about risk factors such as obesity, excessive use of alcohol, the increase in risk of incident AF in patients with RAO may reflect a higher frequency of unmeasured risk factors associated with cardiovascular disease compared to controls and patients with RVO. However, cumulative incidence of death was similar in RVO and RAO patients, thereby implying that the increased risk of AF in RAO – and not RVO – reflects a genuine association independent of differences in health status of cases and controls.

### Clinical implications

This study substantiates the importance of a careful work-up required in patients with retinal vascular occlusion, which is in accordance with other studies. [37, 45] Detection of AF leads to indication for oral anticoagulation (in the presence of at least one CHA_2_DS_2_-VASc point) to efficiently reduce risk of ischemic stroke. [46, 47] Further studies are needed to estimate the effect of oral anticoagulation on the risk of RAO.

### Strengths and limitations

The primary strength of the study is the substantial size of the cohort of patients diagnosed with retinal vascular occlusion in Denmark between 1997 and 2011, and the 1:5 matched population without retinal vascular occlusion.

However, only retinal vascular occlusions diagnosed in hospitals were included, as we did not have access to data regarding patients treated outside of hospitals. As such, it is possible that the identified retinal vascular occlusions treated in hospitals are more severe compared to cases treated outside of hospitals. Consequently, if the association between retinal vascular occlusion and AF is more pronounced for more severe cases, our study may overestimate this association.

## Conclusions

In this nationwide cohort study of patients without previous AF or prior warfarin, we found that retinal vascular occlusions increased the risk of incident AF. In subgroup analyses, the risk was increased in patients with RAO but not in patients with RVO. As retinal vascular occlusions increases the risk of thromboembolism risk in patients with AF, vigilance for incident AF in patients with retinal vascular occlusions is recommended.

## Abbreviation

AF: Atrial fibrillation
ATC: The Anatomical Therapeutic Chemical Classification System
CHA_2_DS_2_-VASc: Congestive heart failure, hypertension, age > 75 years, diabetes, stroke, vascular disease, age 65–75 and female sex score
CI: Confidence interval
HR: Hazard ratio
ICD: International Classification of Diseases
IQR: Inter quartile range IQR
RAO: Retinal artery occlusion
RVO: Retinal vein occlusion
